# An UWB/Vision Fusion Scheme for Determining Pedestrians’ Indoor Location

**DOI:** 10.3390/s20041139

**Published:** 2020-02-19

**Authors:** Fei Liu, Jixian Zhang, Jian Wang, Houzeng Han, Deng Yang

**Affiliations:** 1 School of Environment Science and Spatial Informatics, China University of Mining and Technology (CUMT), Xuzhou 221116, China; pntrc@cumt.edu.cn; 2 National Quality Inspection and Testing Center for Surveying and Mapping Products, Beijing 100830, China; 3 School of Geomatics and Urban Spatial Informatics, Beijing University of Civil Engineering and Architecture (BUCEA), Beijing 102616, China; wangjian@bucea.edu.cn (J.W.); hanhouzeng@bucea.edu.cn (H.H.); yangdeng@stu.bucea.edu.cn (D.Y.)

**Keywords:** monocular vision, UWB, ORB-SLAM, pedestrian indoor location, scale ambiguity

## Abstract

This paper proposes a method for determining a pedestrian’s indoor location based on an UWB (ultra-wideband) and vison fusion algorithm. Firstly, an UWB localization algorithm based on EKF (extended Kalman filter) is proposed, which can achieve indoor positioning accuracy of 0.3 m. Secondly, a method to solve scale ambiguity and repositioning of the monocular ORB-SLAM (oriented fast and rotated brief-simultaneous localization and mapping) algorithm based on EKF is proposed, which can calculate the ambiguity in real time and can quickly reposition when the vision track fails. Lastly, two experiments were carried out, one in a corridor with sparse texture and the other with the light brightness changing frequently. The results show that the proposed scheme can reliably achieve positioning accuracy on the order of 0.2 m; with the combination of algorithms, the scale ambiguity of monocular ORB-Slam can be solved, with the failed vision trace repositioned by UWB, and the positioning accuracy of UWB can be improved, making it suitable for pedestrian location in indoor environments with sparse texture and frequent light brightness changes.

## 1. Introduction

Indoor localization and navigation are considered an enabler for a variety of applications, such as guidance of passengers in airports, conference attendees, and visitors in shopping malls, hospitals, or office buildings [1]. Image-based localization has been studied for a long time in the field of human user indoor localization [2,3], and it can be roughly classified into two groups. In one category, researchers take advantage of the landmarks present in the environment to estimate the camera matrix and extract the query location [4,5]. The other category includes the works that use a stored image database annotated with the position information of the cameras, such as image fingerprinting-based methods [6,7]. In GPS-denied environments, such as underwater and indoor environment, it has been demonstrated that VO (vision odometry) provides relative position errors ranging from 0.1% to 2% [3,8]. Camera-based localization systems have been promoted as promising positioning solutions for applications in the industry, as well as robot and pedestrian localization and navigation [9]. However, there are some challenges for visual localization methods, such as the quality and distinctiveness of the query images, fast motion possibly making the camera-visible scene blurry, and sparse texture, or too bright or too dark optical fiber, which lead to the failure of visual location [10,11]. In the process of essential matrix calculation, the relative displacement between adjacent images is usually normalized, which leads to scale ambiguity of monocular vision. The calculated absolute position and velocity are sensitive to the scale, which should be estimated as to its accuracy [12,13,14]. Ultra-wideband (UWB) technology requires a low energy supply and has good anti-multipath effect, high security, low system complexity, high positioning accuracy, etc., so it is widely used in indoor positioning [15,16,17]. It was demonstrated that the complementary characteristics of these sensors can be exploited to not only solve the problems of visual initialization, scale ambiguity, and absolute spatial benchmark, but also improve the positioning accuracy and frequency of UWB, as well as the potential for active navigation and location, obstacle detection, and real-time transmission of video stream information, which is a feasible fusion location method. There are a few reports in the literature on combining vision and UWB. An indoor location method based on a combination of monocular vision and UWB was proposed which can effectively overcome the problem of monocular vision ORB-SLAM tracking failure and effectively suppress the influence of UWB non-line-of-sight error; the positioning accuracy was able to reach the sub-decimeter level [18]. Enhanced UAV indoor navigation through SLAM-augmented UWB localization was proposed, in which the SLAM-augmented UWB localization had a 90% quantile error of 13.9 cm, and it was shown that the method is capable of providing positioning data to the control system to allow for effective navigation of a drone in the environment. However, in this method, the odometer are used to estimate the altitude, and the flight area map is established through several flights [19]. Ramirez et al. put forward a relative localization method using computer vision and UWB range for a flying robot and showed that the errors in estimated relative positions were between ± 0.190 m on the x-East axis and ± 0.291 m on the z-North axis at the 95% confidence level. In this paper, a computer vision system mounted on the flying robot calculates the relative angle between the nodes, not for the position solution [20]. Benini et al. had studied IMU/UWB/Vision-based EKF for mini-UAV localization in indoor environment, which was based on artificial well-known markers in terms of size and position in the considered environment [21]. Nyqvist et al. showed that UWB can be used to aid visual-inertial simultaneous localization and mapping to obtain improved drift-free global six degree-of-freedom pose estimates [22].

To sum up, in the indoor environment, monocular vision positioning has some problems, such as visual lock being easily lost and scale ambiguity. Through integration with UWB, not only can the above problems be solved, but also higher positioning accuracy can be obtained by fusion. At the same time, there is not much need for UWB base stations.

This paper is organized as follows. In Section 2, the theory of the localization algorithm is first presented, including the UWB positioning algorithm, visual positioning algorithm, and the combined fusion algorithm for UWB and vision. In Section 3, two experiments for the verification of the localization algorithm are carried out, and the positioning results are presented and discussed. Finally, the research contents, methods, and future improvements of the paper are summarized and analyzed.

## 2. Methodology

**Figure 1:**
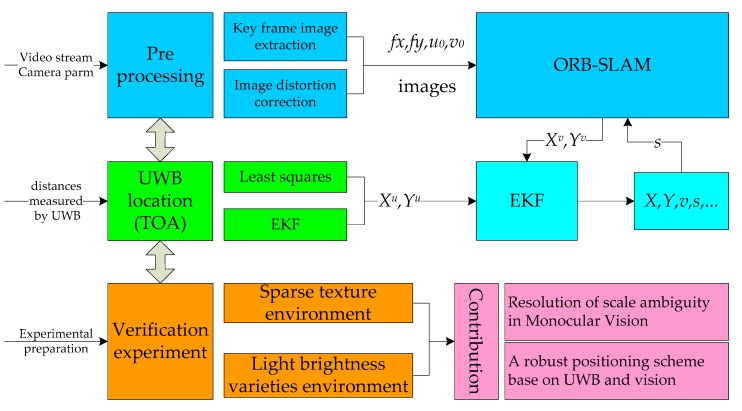
The technology roadmap.

In the indoor environment, due to the single wall texture and the change of light brightness, it is easy to fail vision SLAM. Moreover, the SLAM technology based on monocular camera has some problems, such as scale ambiguity, axis offset etc. In order to solve those problems, we study a pedestrian positioning technique, mainly including space datum construction based on UWB, research of the visual positioning algorithm, a fusion algorithm for UWB and vision, and verified experiments. The technology roadmap is shown in Figure 1, and the details are as follows.

Firstly, an UWB localization algorithm based on EKF is put forward. Secondly, a visual positioning scheme based on ORB-SLAM is adopted [23,24], including the preprocessing of the video stream and the epipolar geometry constraint. The former is composed of key frame image extraction and image distortion correction [25,26]. Thirdly, an EKF fusion algorithm is constructed which combines the UWB and visual positioning data; in particular, the scale ambiguity problem of monocular vision is solved. Then, the pedestrian position is acquired. Finally, through two tests of a corridor with sparse texture and a room with frequently changing light brightness, the UWB indoor positioning method and its accuracy, as well as the coupled UWB and vision algorithm and its positioning accuracy, are verified.

The main contributions and innovations are the resolution of scale ambiguity and axis offset in monocular vision in real time and a robust positioning scheme for vision SLAM that UWB positioning results provide an absolute benchmark for visual positioning, and when visual positioning fails, UWB is used to quickly restore visual positioning.

### 2.1. The UWB Positioning Algorithm

The TOA [27] (Time of Arrival) positioning method is mainly based on the measurement of the arrival time of the received signal between the anchors and the tag, which is then converted to a distance for positioning. In order to solve the positioning problem of UWB, a nine-dimensional state vector based on EKF is used, which is shown as follows:

(1) $${\hat{x}}_{k}\;=\;\left[\begin{array}{ccc} \Delta x & \Delta \dot{x} & \Delta \ddot{x} \end{array}\begin{array}{ccc} \Delta y & \Delta \dot{y} & \Delta \ddot{y} \end{array}\begin{array}{ccc} \Delta z & \Delta \dot{z} & \Delta \ddot{z} \end{array}\right]$$

where Δx,Δy,Δz are the positions,$\;\Delta \dot{x},\Delta \dot{y},\Delta \dot{z}$ are the velocities, and$\;\Delta \ddot{x},\Delta \ddot{y},\Delta \ddot{z}$ are the accelerations in the X, Y, and Z directions. 

As the system equation and the observation equation are nonlinear, an EKF is adopted to realize a linear approximation of the nonlinear system. It is assumed that the nonlinear system is expressed as [28,29]

(2) $$x_{k}\;=\;f_{k-1}\left(x_{k-1}\right)+w_{k}\;w_{k}\sim N\left(0,Q_{k}\right)$$

(3) $$z_{k}\;=\;h_{k}\left(x_{k}\right)+v_{k}\;v_{k}\sim N\left(0,R_{k}\right)$$

where $x_{k}$ is the state vector of time k; $z_{k}$ is the observation vector of time k; $w_{k}$ and $v_{k}$ are random noise; $f_{k-1}\left(•\right)$ and $h_{k}\left(•\right)$ are the state transition function and transfer function, respectively; and $Q_{k}$ and $R_{k}$ are the system dynamic noise variance matrix and observed noise variance matrix, respectively [28]. The state prediction function is as follows:

(4) $${\hat{x}}_{k}\left(-\right)\;=\;f_{k-1}\left({\hat{x}}_{k-1}\left(+\right)\right)$$

(5) $${\hat{z}}_{k}\;=\;h_{k}\left({\hat{x}}_{k}\left(-\right)\right).$$

$f_{k-1}\left(•\right)$ and $h_{k}\left(•\right)$ can be linearized via Taylor series expansion:

(6) $$\Phi_{k-1}\;\approx \;{\frac{\partial f_{k}}{\partial x}|}_{x\;=\;{\hat{x}}_{k-1}\left(-\right)}$$

(7) $$H_{k}\;\approx \;{\frac{\partial h_{k}}{\partial x}|}_{x\;=\;{\hat{x}}_{k}\left(-\right)}.$$

The prediction covariance matrix is:

(8) $$P_{k}\left(-\right)\;=\;\Phi_{k-1}^{\;}P_{k-1}\left(+\right){\Phi_{k-1}}^{T}+Q_{k-1}.$$

The EKF gain matrix is:

(9) $${\bar{K}}_{k}\;=\;P_{k}\left(-\right){H_{k}}^{T}\;{\left[H_{k}^{\;}P_{k}\left(-\right){H_{k}}^{T}+R_{k}\right]}^{-1}.$$

The state estimate update is:

(10) $${\hat{X}}_{k}\;=\;{\hat{X}}_{k}^{-}+{\bar{K}}_{k}\left(Z_{k}-H\left(k,{\hat{X}}_{k}^{-}\right)\right).$$

The error covariance update is:

(11) $$\;P_{k}\;=\;\left(I-G_{k}H_{k}\right)P_{k}^{-}.$$

### 2.2. The Visual Positioning Algorithm 

Video cameras are sensors that provide rich information about the surrounding world. A camera maps points from the 3D world to 2D images, so we can use those images to infer the locations of objects in the environment [30].

**Figure 2:**
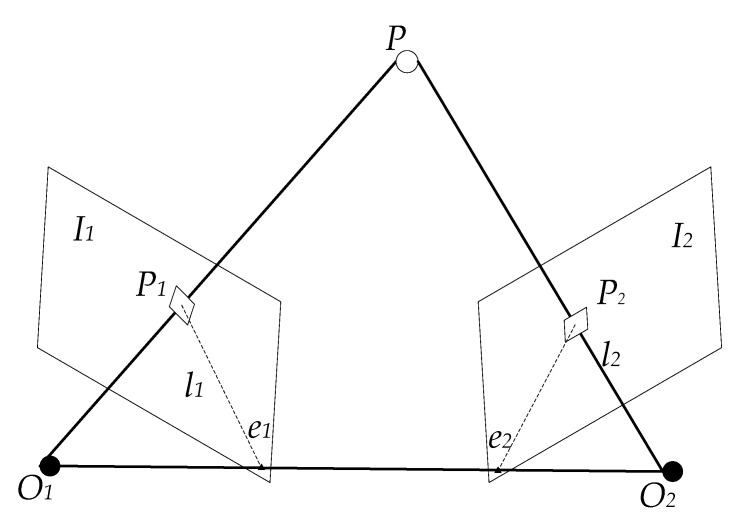
Epipolar geometry constraints.

As shown in Figure 2, let *I_1_* and *I_2_* be two frames of images, *O_1_* and *O_2_* be the exposing positions of the camera where the images were obtained, and *P* be a spatial point such that line *O_1_P_1_* and line *O_2_P_2_* intersect at point *P*. The plane composed of *O_1_*, *O_2_*, and point *P* is an epipolar plane. *O_1_O_2_* is the baseline. The intersection points of line *O_1_O_2_* and image planes *I_1_* and *I_2_*, denoted *e_1_* and *e_2_*, respectively, are epipoles. The intersections of the epipolar plane and image planes *I_1_* and *I_2_* are denoted *L_1_* and *L_2_*, respectively, and are epipolar lines. $\;p_{1}\;$and $p_{2}$ are the intersections of the image planes *I_1_* and *I_2_* and the spatial point *P*, respectively. The motion from the first frame to the second frame is denoted R,t [26,31].

**Figure 3:**
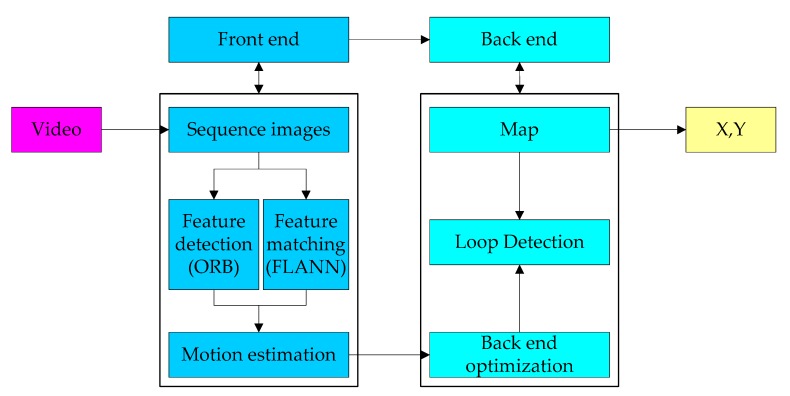
A rough flowchart of oriented fast and rotated brief-simultaneous localization and mapping (ORB-SLAM).

Figure 3 shows a rough flowchart of ORB-SLAM, and there are two important processing courses: Front end and back end. The main tasks of the front end are estimating the motion between adjacent images and building a local map. The image feature detection and description are carried out via ORB [32], and the image matching method is FLANN (fast library for approximate nearest neighbors) [33], which are not described in detail here. Some of the details are given in the following.

Suppose that in the first frame of the image coordinate system, the spatial position of point *P* is:

(12) $$P\;=\;{\left[X_{G},Y_{G},Z_{G}\right]}^{T}$$

According to the pinhole camera model, the pixel coordinates of$\;p_{1}\;$and $p_{2}$ are:

(13) $$p_{1}\;=\;KP,\;\;p_{2}\;=\;K\left(RP+t\right)$$

where *K* is the camera internal parameter. 

We assume that:

(14) $$x_{1}\;=\;K^{-1}p_{1},x_{2}\;=\;K^{-1}p_{2}$$

where $x_{1}$ and $\;x_{2}$ are the normalized plane coordinates of two pixels; when these are substituted into the above formula, the following results are obtained:

(15) $$x_{2}\;=\;Rx_{1}+t$$

We multiply both sides by t^.

(16) t^x2 = t^Rx1

Then, we multiply both sides by $x_{2}^{T}$ to get:

(17)  x2Tt^x2 = x2Tt^Rx1

where t^x2 is a vector perpendicular to both t and $x_{2}$. Therefore, the above formula can be simplified as follows:

(18) x2Tt^Rx1 = 0

Substituting $\;p_{1}$,$p_{2}$ back in, we get:

(19) p2TK−Tt^RK−1p1 = 0

The above two formulas become epipolar constraints, including translation and rotation, and the middle parts are recorded as two matrices—Fundamental Matrix F and Essential matrix E—to further simplify the polar constraint:

(20) E = t^R,F = K−TEK−1,x2TEx1 = p2TFp1 = 0

The spatial position relationship of two synonymous points is given for the epipolar constraint, so the camera pose estimation problem becomes the following two steps: The calculation of E or F according to the pixel coordinates of the synonymous point, and the calculation of the R,t base on E or F.

Aside from the Fundamental Matrix F and Essential matrix E, the Homography H [24] can also be used to estimate the motion when the feature points are on the same plane, such as a wall or the ground.

In addition, the PnP (perspective-n-point) [34] algorithm also can be used to estimate the motion after initialization of the monocular odometer, and it does not need the epipolar geometry constraint. 

The back end includes the optimization, loop detection, and mapping. The back end accepts the camera position and attitude measured at different times, as well as the loop detection information, and optimizes it, then constructs globally consistent trajectories and maps. The details can be seen in the literature [23,24].

### 2.3. The Fusion Positioning Algorithm

Following the method in [35], the state-space models are:

(21) $$\;X_{k+1}\;=\;\varphi X_{k}+w_{k}\;w_{k}\sim N\left(0,Q_{k}\right)$$

(22) $$Z_{k}\;=\;HX_{k}+\tau_{k\;}\tau_{k}\sim N\left(0,R_{k}\right)$$

where $w_{k}$ and $\tau_{k}$ are the independent, zero mean. Gaussian noise processes of covariance matrices $Q_{k}$ and $R_{k}$, respectively. Further,

(23) $$X\;=\;{\left[\begin{array}{ccc} x & y & v \end{array}\;\;\begin{array}{cc} \theta & \begin{array}{cc} s & \emptyset \end{array}\;\; \end{array}\right]}^{T}$$

where x, y represent the plane coordinates, v represents the velocity of the pedestrian, θ represents the movement heading angle, s represents the scale ambiguity, and ∅ represents the deflection angle between the plane coordinates calculated by vision and the plane coordinates calculated by UWB.

According to the error equation of vision and UWB, the corresponding state model is:

(24) $${\tilde{X}}_{k+1}\;=\;\left[\begin{array}{c} \begin{array}{ccc} 1 & 0 & sin\theta \\ 0 & 1 & cos\theta \\ 0 & 0 & 1 \end{array}\;\;\begin{array}{ccc} 0 & 0 & 0\\ 0 & 0 & 0\\ 0 & 0 & 0 \end{array}\\ \begin{array}{ccc} 0 & 0 & 0\\ 0 & 0 & 0\\ 0 & 0 & 0 \end{array}\;\;\begin{array}{ccc} 1 & 0 & 0\\ 0 & 1 & 0\\ 0 & 0 & 1 \end{array} \end{array}\right]X_{k}$$

Among the variables, $w_{x}$ and *W_y_* represent the plane position error, while $w_{v}$, $w_{\theta \;},$ and $w_{s}\;$represent the velocity error, heading angle error, and visual scale factor error, respectively. 

If the position and course measured by vision and the position measured by UWB are taken as observations, the observation equation of integrated navigation can be expressed as follows:

(25) $$\left[\begin{array}{c} X_{uwb}\\ Y_{uwb} \end{array}\right]\;=\;\left[\begin{array}{ccc} 1 & 0 & 0\\ 0 & 1 & 0 \end{array}\;\;\begin{array}{ccc} 0 & 0 & 0\\ 0 & 0 & 0 \end{array}\right]X+e_{uwb}$$

(26) $$\left[\begin{array}{c} X_{vision}\\ Y_{vision} \end{array}\right]\;=\;\left[\begin{array}{ccc} cos\emptyset & -sin\emptyset & 0\\ sin\emptyset & cos\emptyset & 0 \end{array}\;\;\begin{array}{ccc} 0 & X_{vision} & 0\\ 0 & Y_{vision} & 0 \end{array}\right]X+e_{vision}$$

$X_{vision}$ and $Y_{vision}$ represent the plane position measured by the vision sensor, and $X_{uwb}$ and $Y_{uwb}$ represent the plane position measured by UWB. $e_{vision}$ represents the position measurement error of the vision sensor, while $e_{uwb}$ represents the UWB position measurement error.

Although $\varphi \left(k,X_{k}\right)$ is a nonlinear matrix, this problem can be solved effectively by expansion of the first-order Taylor Series. We define:

(27) $$\varphi_{k+1,k}\;=\;{\frac{\partial \varphi \left(k,X\right)}{\partial X}|}_{X\;=\;X_{k}}\;,$$

(28) $$H_{k}\;=\;{\frac{\partial H\left(k,X\right)}{\partial X}|}_{X\;=\;X_{k}^{-}}\;.$$

The state estimate propagation is:

(29) $$\;{\hat{X}}_{k}^{-}\;=\;\varphi (k,{\hat{X}}_{k-1}^{-})$$

The error covariance propagation is:

(30) $$P_{k}^{-}\;=\;\varphi_{k,k-1}P_{k-1}\varphi_{k,k-1}^{T}+Q_{k-1}$$

The Kalman gain matrix is:

(31) $$\;G_{k}\;=\;P_{k}^{-}H_{k}^{T}{\left[H_{k}P_{k}^{-}H_{k}^{T}+R_{k}\right]}^{-1}$$

The state estimate update is:

(32) $${\hat{X}}_{k}\;=\;{\hat{X}}_{k}^{-}+G_{k}\left(Z_{k}-H\left(k,{\hat{X}}_{k}^{-}\right)\right)$$

The error covariance update is:

(33) $$\;P_{k}\;=\;\left(I-G_{k}H_{k}\right)P_{k}^{-}$$

## 3. Experimental Verification

### 3.1. Introduction of the Experimental Device

**Figure 4:**
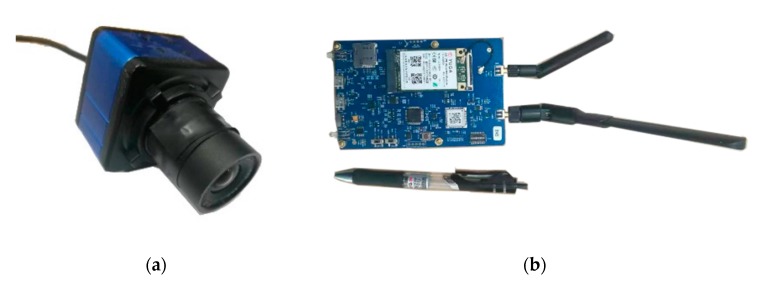
Experiment devices. (**a**) Camera; (**b**) ultra-wideband (UWB) anchor and tag two-in-one device.

A camera and UWB equipment are the main experimental devices in this study, as shown in Figure 4. The camera, named Guardian, can be bought from Taobao.com. Its performance indicators are shown in Table 1. The UWB equipment is a self-developed piece of equipment with UM100 module of Shanghai upositon Co., Ltd., the technical indicators of which are shown in Table 2. In the process of the experiment, original images were collected by the camera and used to calculate the motion trajectory. The distance between the tag and the anchors was obtained by UWB tag and then used to calculate the positioning information.

The UWB anchor and tag can be used not only as an anchor device, but also as a tag device, and can change work mode automatically. The ranging accuracy of the UWB chip is about 10 cm, and the positioning accuracy can reach 15 ~ 30 cm [36].

### 3.2. Experiment 1

**Figure 5:**
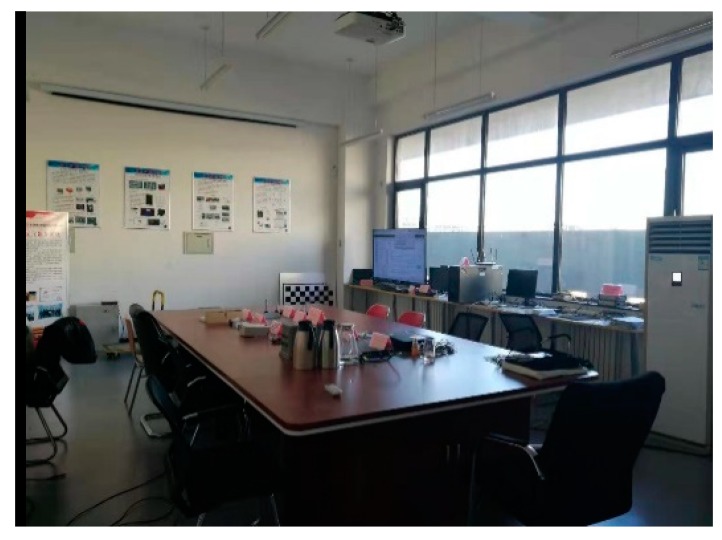
The test environment of experiment 1.

This experiment was carried out in a laboratory, shown in Figure 5. As the brightness adjustment mode of the camera is automatic, when the camera faces the window, the brightness of the image will be reduced, especially in the process of turning. On the contrary, when it faces the other side, the brightness will be increased. Frequent changes in the brightness lead to ORB-SLAM tracking failure. Four UWB anchors were deployed at the four corners of the room, and a test route was designed. The experiment data were collected along the route with three loops in total.

**Figure 6:**
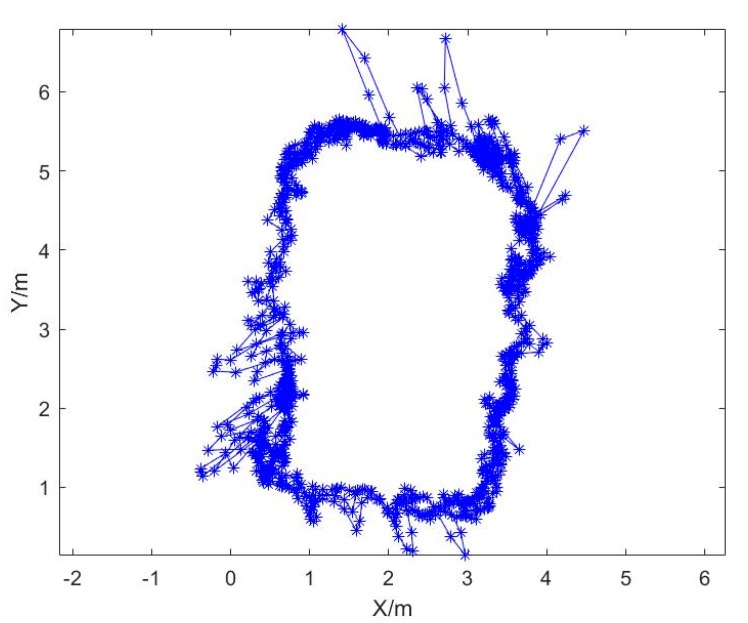
The UWB positioning results.

Figure 6 shows the UWB positioning results. It can be seen that a great part of the results was consistent with the designed route, but there were also big differences between the results and the actual route, such as in the left and bottom corner and along the top line.

**Figure 7:**
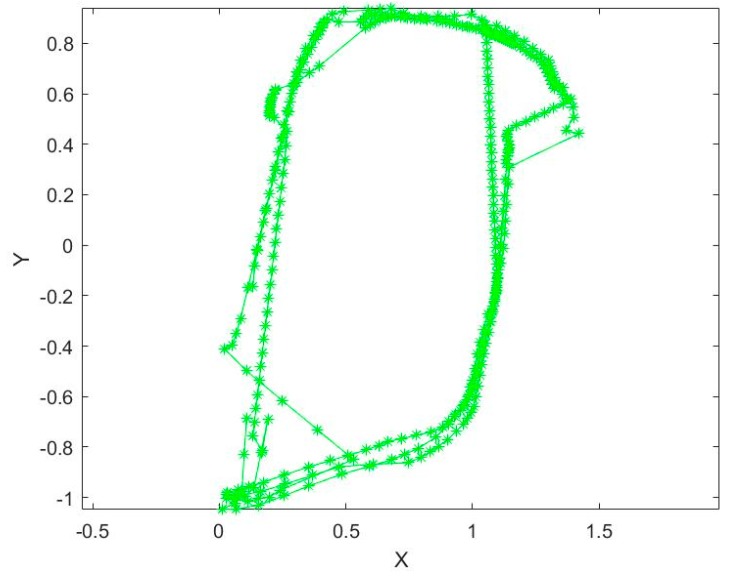
The vision raw positioning results.

Figure 7 shows the vision raw positioning results, from which the following can be seen: First, some of the results describe the walking trajectory accurately; secondly, there is a scale ambiguity problem due to the monocular method; thirdly, positioning failure phenomena appeared many times due to the sparse texture, brightness changes, in situ turns, and other factors; and last in the processing course, although the positioning results were improved by the loop detection and correction of ORB-SLAM, there are big errors in the right and top corner.

From Figure 6 and Figure 7, we can see that UWB can solve the positioning problem for a pedestrian in indoor situations; however, there are major location errors. The vision positioning method can achieve accurate location results in a small area, but it will be affected by many factors, such as the texture, light brightness, etc.

**Figure 8:**
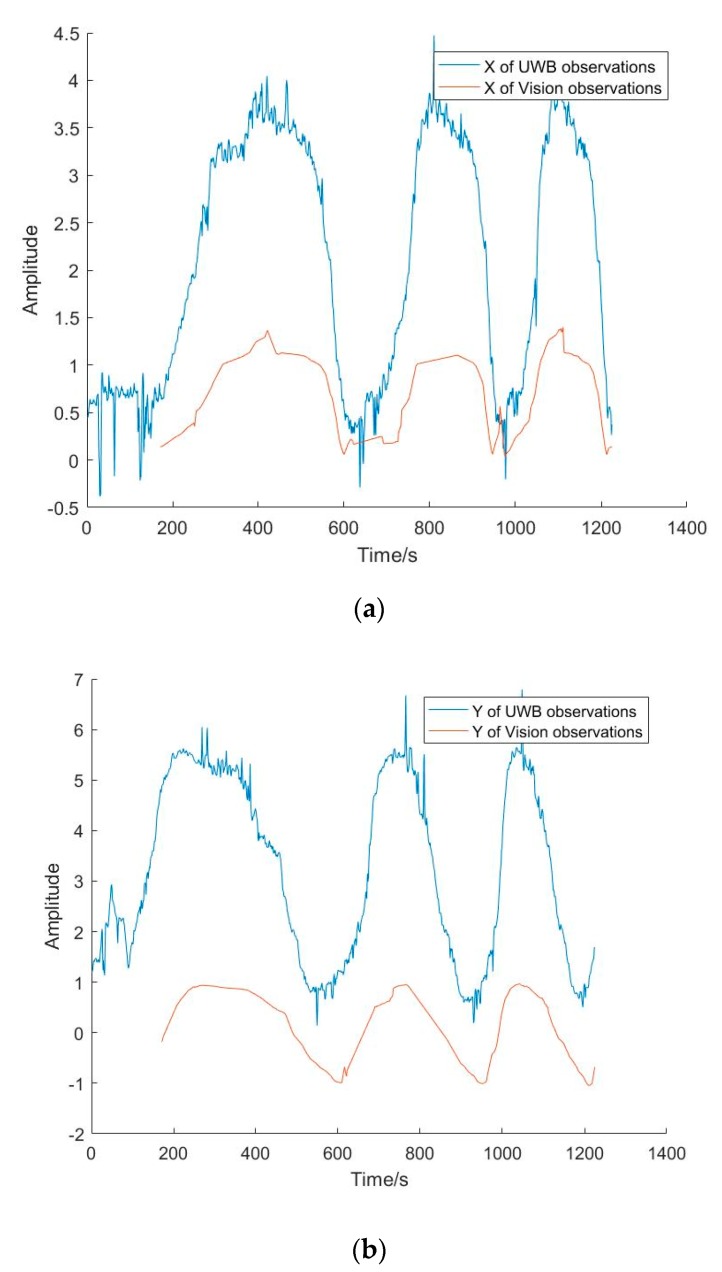
The consistency between the UWB and vision observations. (**a**) Comparison of synchronization of observations in X direction; (**b**) Comparison of synchronization of observations in Y direction

Figure 8 shows the consistency between the UWB and vision observations. We can see that the trends of the amplitudes of the UWB and vision positioning results are consistent, except that the vison positioning results for the beginning 180 s are missing because of the initial influence of ORB-SLAM. This shows that the synchronization achieved between the UWB and vision sensors with the computer time is good. In terms of smoothness, the curve of vision observations is better than that of UWB, which illustrates that the vision positioning accuracy is better than that by UWB.

**Figure 9:**
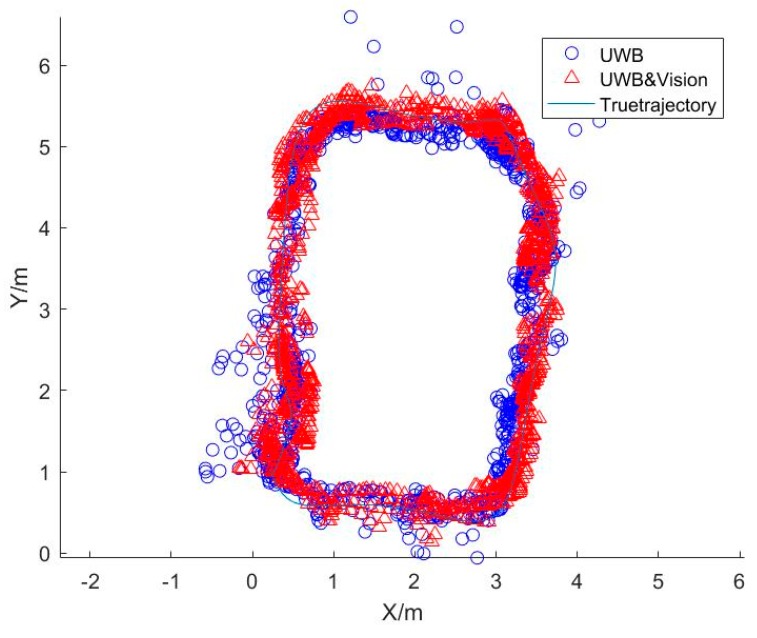
The positioning results.

**Figure 10:**
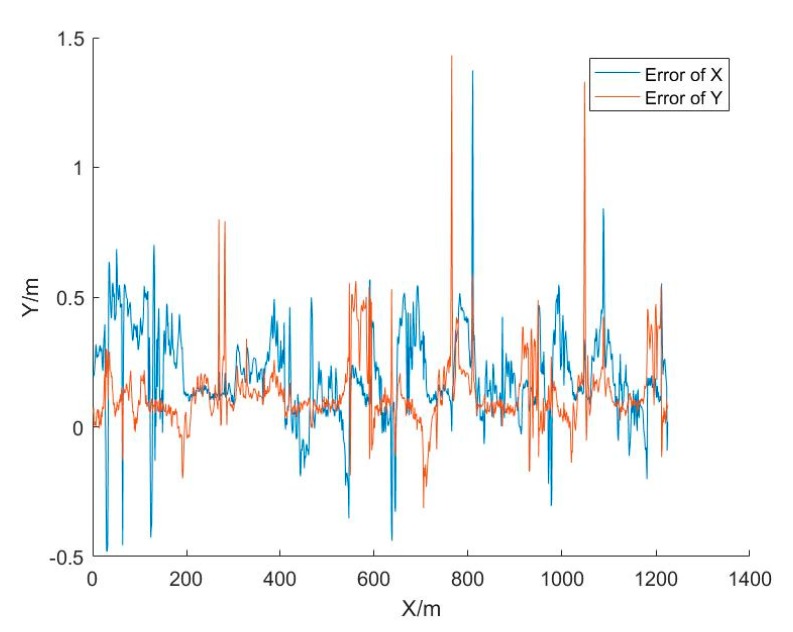
The UWB positioning errors.

**Figure 11:**
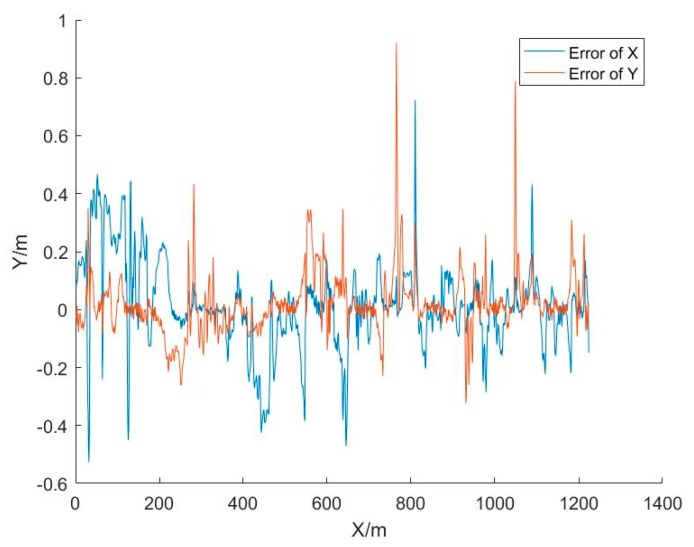
The positioning errors of the UWB and vision combination.

Figure 9 shows the positioning results, where the blue circles represent the positioning results of UWB, the red triangles are the location points calculated by the combination of UWB and vision, and the blue line is the actual trajectory. It can be seen that almost all red points are distributed at both sides of the actual route, indicating that the combined algorithm’s results are more accurate than those of UWB alone. In addition, the vision position errors which occurred in Figure 7 are limited by the coupled algorithm, and the scale ambiguity is solved too. The positioning errors of UWB and the coupled method are shown in Figure 10 and Figure 11.

Figure 10 shows the difference between the UWB positioning results and the actual route. The maximum and minimum errors in the X direction were 1.37 and 0 m, respectively. The maximum and minimum errors in the Y direction were 1.43 and 0 m, respectively. The RMSE (root mean square error) of the plane error was 0.32 m, as shown in Table 3.

Figure 11 shows the difference between the positioning results of the UWB and vision combination and the actual route. The maximum and minimum errors in the X direction were 0.72 and 0 m, respectively. The maximum and minimum errors in the Y direction were 0.92 and 0 m, respectively. The RMSE of the plane error was 0.18 m, as shown in Table 3. From Table 3 and Table 4, we can see that the positioning accuracy of the coupled method is higher than that of UWB alone by about 43.75%.

### 3.3. Experiment 2

**Figure 12:**
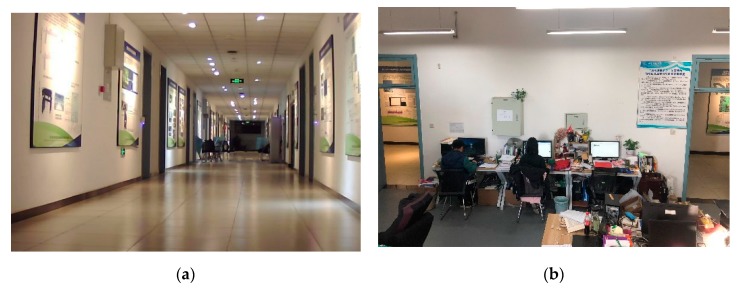
The scene of experiment 2. (**a**) Corridor; (**b**) laboratory room.

Experiment 2 was carried out in a corridor and laboratory room. The length of the corridor is about 65 m, and the width is about 3 m. The area of the laboratory room is about 6 × 8 m, shown in Figure 12. It can be seen that the texture of the corridor is sparse.

**Figure 13:**
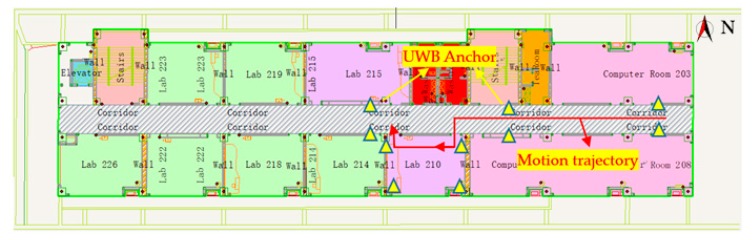
Schematic diagram of the location of the UWB base stations and rough walking route.

In this experiment, a total of 10 UWB anchors were deployed in a narrow corridor and a rectangular laboratory, and the approximate locations are shown in Figure 13. On the indoor map, N represents the north direction. Since the base image is a picture and can be scaled at will, the true coordinates of the anchors were obtained by the electronic total station, with positioning accuracy at the centimeter level. In addition, the figure shows the general test route of the experiment.

**Figure 14:**
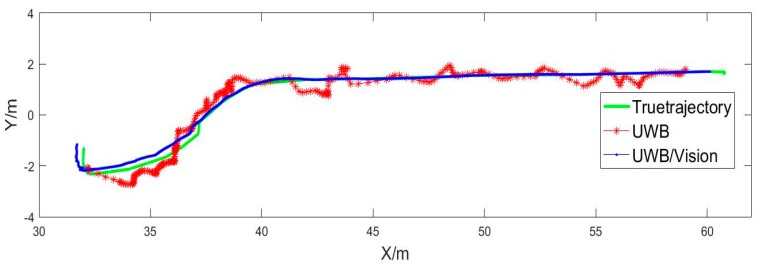
The positioning results.

As we can see from Figure 14, the positioning results calculated by UWB alone and by UWB/Vision in combination are in good agreement with the actual trajectory. In addition, UWB can be used as the initial positioning parameter of the vision method in the combination process, and after vision positioning failure, the positioning can be restored in situ, and then the problem of continuous positioning can be solved.

Since the accurate position at each sampling time could not be accurately recorded in the process of the experiment, in order to verify the positioning accuracy of the two methods, we interpolated the walking route according to a certain number of points. Then, the calculation results of the two methods were compared with the nearest points on the route, and the positioning accuracy of the two methods was thus obtained.

**Figure 15:**
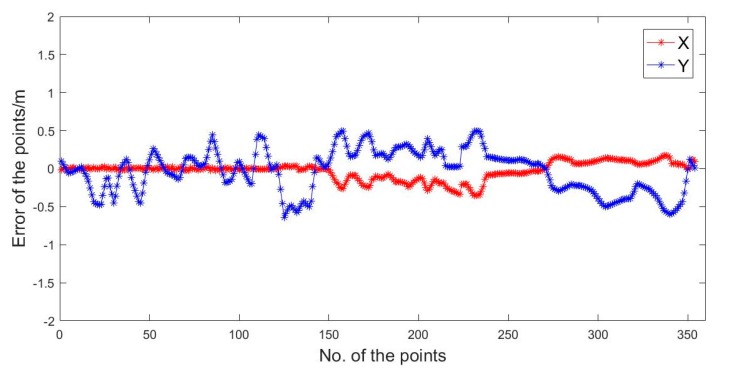
The UWB positioning errors.

In Figure 15 and Table 4, we can see the positioning results solved by UWB. In the X direction, the maximum error was −0.35 m. In the Y direction, the maximum error was −0.64 m, and the RMSE was ± 0.31 m.

**Figure 16:**
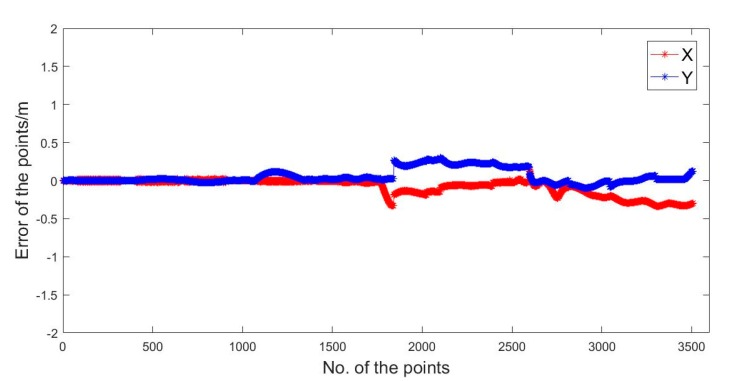
The UWB/Vison combination positioning errors.

In Figure 16 and Table 4, we can see the positioning results solved by UWB/Vision. In the X direction, the maximum error was −0.33 m. In the Y direction, the maximum error was 0.30 m, and the RMSE was ± 0.17 m.

**Figure 17:**
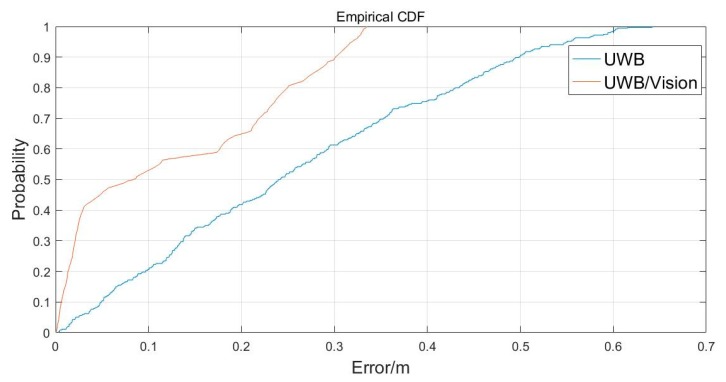
The cumulative distributions of plane errors.

The blue line in Figure 17 is the positioning accuracy CDF (cumulative distribution function) curve of UWB. It can be seen that 20%, 22%, 20%, 13%, 15%, and 10% of the points had accuracy of the order of 0.1, 0.1–0.2, 0.2–0.3, 0.3–0.4, 0.4–0.5, and 0.5–0.7 m, respectively. The red line is the positioning accuracy CDF curve of UWB/Vison in combination. It can be seen that about 53%, 12%, 25%, and 10% of the points had accuracy of the order of 0.1, 0.1–0.2, 0.2–0.3, and 0.3–0.4 m, respectively.

Through the study of this paper, we can see that SLAM technology, as a top positioning and mapping method, has been widely studied in indoor positioning, and can be used for navigation while obtaining indoor information. However, in the indoor environment, due to the single wall texture and the change of light brightness, it is easy to fail in feature extraction and matching, resulting in the interruption of data processing, as shown in Figure 7. Moreover, the SLAM technology based on monocular camera has some problems, such as scale ambiguity, axis offset, etc.

UWB is a kind of pulse radio technology with high bandwidth ratio. It usually uses an ultrashort pulse (or impulse pulse) to generate ultra-wideband information signal, which has the characteristics of high ranging accuracy, good stability, low power consumption, and good resistance to multipath. UWB positioning technology based on the TOA principle is widely used in submeter precision indoor positioning. However, due to the influence of indoor building pattern, decoration, and personnel movement, there are serious non-line-of-sight measurement environments, which lead to the decline of ranging accuracy. In addition, affected by non-line-of-sight, ranging range, diversified spatial pattern and other factors, there are high requirements for the number and location of UWB anchor.

First of all, the scale ambiguity and axis offset in monocular SLAM technology are solved as unknown parameters. Secondly, in view of the location failure caused by sparse texture or light change, the UWB measurement results are used as observations to assist the restoration of the absolute positioning reference of SLAM in real time. In terms of positioning accuracy, unlike VO technology, it can only provide relative position errors ranging from 0.1% to 2% [3,8]. This method not only solves the problem of absolute positioning of monocular vision, but also achieves the same absolute positioning accuracy as that of literature [19,20]. In the process of indoor positioning, we can accurately determine which side of the wall pedestrians are located, and obtain the environmental information of pedestrians, which plays a good role in emergency rescue in dangerous situations such as nursing homes or elderly people living alone. Then, if the pedestrian is still, the vision-based positioning technology will not produce a large error drift such as the micro-electro-mechanical system- inertial navigation systems (MEMS-INS) positioning technology, and can achieve an absolute positioning accuracy of the order of magnitude better than 10^−3^ m [26], and is not limited by the rest time. Vision belongs to a passive positioning method, and can achieve 10–60 Hz or even higher sampling frequency. Therefore, the fusion technology of vision and UWB can reduce the number of anchors of UWB, reduce the workload, save time, and improve the positioning frequency. 

The loose combination algorithm used in the research process of this paper cannot take the ranging information of UWB and the results of visual feature extraction and matching as observations, so it cannot achieve UWB-assisted visual fast search of homonym points, and real-time recovery of SLAM initialization when visual positioning fails. In addition, the position change information of vision measurement is not fused with UWB ranging information, which cannot effectively assist UWB to improve the ranging accuracy in non-line-of-sight environment. Moreover, affected by factors such as image resolution and frame rate, as well as the performance of computer hardware, there is still great potential for optimization in real-time processing. In a word, the deep fusion and efficient processing of vision and UWB data will be the focus of our research in the future.

## 4. Conclusions

Vision sensors as a streaming media technology can not only achieve rich texture information but can also be transmitted in real time. If used in location methods, they can not only obtain the localization of people but can also know the surrounding information, which is very suitable for indoor location of a pedestrian. However, at present, indoor positioning methods based on vision fail due to sparse textures, light that is too bright or too dark, and other factors. In order to solve this problem, an indoor location method based on UWB/Vision combination was proposed in this paper. First of all, an UWB localization algorithm based on EKF was proposed, and the experimental results showed that the algorithm can achieve indoor positioning accuracy of the order of 0.3 m. Secondly, an UWB/Vision fusion location algorithm based on EKF was proposed, and the experimental results showed that the algorithm can achieve indoor location accuracy of the order of 0.2 m, which can tell whether pedestrians are inside or outside the room. The conclusions of this research are as follows:

(1) High relative positioning accuracy can be obtained by using monocular SLAM for indoor positioning, but there is a problem of spatial scale uncertainty and location failures due to factors such as light changes and texture sparsity.
(2) In view of the complex indoor environment, when using pure UWB technology for positioning, there are higher requirements in terms of the number and location of UWB base stations, and it is necessary to measure the coordinates of the base stations in advance.
(3) With the combination of UWB and vision, the scale ambiguity of monocular Slam can be solved. For sparse texture, light brightness variations, and other environmental properties, the vision position method can be repaired using UWB when it fails to locate. The local positioning results of vison are more accurate, which can be used to improve the accuracy of UWB.
(4) For an integrated environment of an indoor corridor and a room, the number of UWB base stations can be reduced. For example, base stations can be set up in corridors for initial positioning or initialization work after positioning failure. This solves the problem of positioning in the room via vision.
(5) There are also challenges in the combination of UWB and Vison, for example, the high time cost and higher frequencies of the initial problem. As the algorithms are coupled in a loose way, the UWB observations cannot be used to directly assist image matching, etc. Therefore, it is necessary to research a tightly coupled algorithm for UWB and vision positioning in the future.

With the rapid development of urbanization, the demand for emergency rescue in indoor or urban underground space is increasing. Through the combination of vision and UWB, not only can the emergency location of indoor rescue workers be realized, but also the video data of the rescue scene can be obtained in real time, which has great application potential.

## Figures and Tables

**Table 1:** Performance of the camera.

Performance	Parameter
Frame rate (FPS)	10
Fx (pixels)	3637.74
Fy (pixels)	3658.25
Resolution (pixels)	1920 × 1080
Brightness mode	Auto

**Table 2:** Performance of the UWB anchor and tag two-in-one device [36].

Performance	Parameter
Size	9 × 12.5 × 0.7 cm
Operating voltage	3–5 V
Receiving sensitivity	−118 dBm
Ranging accuracy	≤ 10 cm
Line-of-sight ranging distance	Max 880 m
Positioning accuracy	≤ 30 cm
Positioning sampling rate	1–5 Hz

**Table 3:** Root mean square error (RMSE) values of the positioning accuracy of UWB alone and UWB/Vision.

Sensor	UWB	UWB/Vision
RMSE[m]	0.32	0.18

**Table 4:** RMSE values of the positioning accuracy of UWB alone and UWB/Vision.

Sensor	UWB	UWB/Vision
RMSE[m]	0.30	0.17
